# Cell necrosis, intrinsic apoptosis and senescence contribute to the progression of exencephaly to anencephaly in a mice model of congenital chranioschisis

**DOI:** 10.1038/s41419-019-1913-6

**Published:** 2019-09-26

**Authors:** Marc Oria, Soner Duru, Rebeca L. Figueira, Federico Scorletti, Lucas E. Turner, Irati Fernandez-Alonso, Alejandra Fernandez-Martin, Mario Marotta, Lourenco Sbragia, Aimen F. Shaaban, Jose L. Peiro

**Affiliations:** 1 Center for Fetal and Placental Research, Cincinnati Children’s Hospital Medical Center (CCHMC), Cincinnati, OH USA; 2 Laboratory of Experimental Fetal Surgery “Michael Harrison”, Division of Pediatric Surgery, Department of Surgery and Anatomy, Ribeirao Preto Medical School, University of Sao Paulo-USP, Ribeirao Preto, Brazil; 3 Department of Pediatric Surgery, Hospital Bambino Gesu, Rome, Italy; 4 The Chicago Institute for Fetal Health, Ann & Robert H. Lurie Children’s Hospital of Chicago, Chicago, IL USA; 5 Department of Pediatric Surgery, Northwestern University, Feinberg School of Medicine, Chicago, IL USA; 6 Bioengineering, Cell Therapy and Surgery in Congenital Malformations Laboratory, Vall d’Hebron Research Institute (VHIR), Universitat Autònoma de Barcelona, Barcelona, Spain

**Keywords:** Disease model, Neural tube defects

## Abstract

Exencephaly/anencephaly is one of the leading causes of neonatal mortality and the most extreme open neural tube defect with no current treatments and limited mechanistic understanding. We hypothesized that exencephaly leads to a local neurodegenerative process in the brain exposed to the amniotic fluid as well as diffuse degeneration in other encephalic areas and the spinal cord. To evaluate the consequences of in utero neural tissue exposure, brain and spinal cord samples from E17 exencephalic murine fetuses (maternal intraperitoneal administration of valproic acid at E8) were analyzed and compared to controls and saline-injected shams (*n* = 11/group). Expression of apoptosis and senescence genes (p53, p21, p16, Rbl2, Casp3, Casp9) was determined by qRT-PCR and protein expression analyzed by western blot. Apoptosis was measured by TUNEL assay and PI/AV flow cytometry. Valproic acid at E8 induced exencephaly in 22% of fetuses. At E17 the fetuses exhibited the characteristic absence of cranial bones. The brain structures from exencephalic fetuses demonstrated a loss of layers in cortical regions and a complete loss of structural organization in the olfactory bulb, hippocampus, dental gyrus and septal cortex. E17 fetuses had reduced expression of NeuN, GFAP and Oligodendrocytes in the brain with primed microglia. Intrinsic apoptotic activation (p53, Caspase9 and 3) was upregulated and active Caspase3 localized to the layer of brain exposed to the amniotic fluid. Senescence via p21-Rbl2 was increased in the brain and in the spinal cord at the lamina I-II of the somatosensory dorsal horn. The current study characterizes CNS alterations in murine exencephaly and demonstrates that degeneration due to intrinsic apoptosis and senescence occurs in the directly exposed brain but also remotely in the spinal cord.

## Introduction

Exencephaly is the most severe form of all neural tube defects (NTD) with an incidence of 3 every 10,000 pregnancies^1^. The neurulation process occurs during the fourth week of human gestation and disrupted closure and absence of calvaria result in exencephaly. The unprotected fetal brain tissue undergoes progressive damage with advancing gestational age due to exposure to chemical and mechanical factors in the intrauterine environment^2,3^. In this severe form of neural tube defect the brain is totally disorganized with few defined structures^4^ and some functions are always present^5,6^. Exencephaly becomes anencephaly during gestation due this aggressive environment and the brain tissue can be destroyed by 8−10 weeks of gestation^7^. Acrania-exencephaly-anencephaly sequence theory was proposed by the observation of exencephalic fetuses during pregnancy and the later delivered with anencephaly^8^; this theory suggests a “peeling off” of the neural tissue which has been demonstrated in mice at E18.5 ^9^.

This malformation, incompatible with extrauterine life and currently untreatable, is poorly investigated and the degenerative mechanisms in the brain and the spinal cord are not clearly defined.

The use of an exencephaly mice model, induced following maternal administration of the antiepileptic drug Valproic Acid (VPA)^10,11^, in the current study allows characterization of the neural damage present in the brain and distal spinal cord of exencephalic fetuses to elucidate the degenerative mechanisms involved in the pathogenesis of exencephaly before anencephaly occurs.

## Material and methods

All experimental protocols were approved by the Institutional Animal Care and Use Committee at The Children’s Hospital of Cincinnati and followed guidelines set forth in the National Institutes of Health Guide for Care and Use of Laboratory Animals (IACUC 2013-0293).

### Animal preparation and valproic acid (VPA) exposure

Timed-pregnant CD1 mice weighing 30−35 g (Charles River Laboratories, Inc, Wilmington, MA), were individually housed with a standard dark:light schedule (10:14; 22 °C) and access to water and standard chow ad libitum. Valproic acid (VPA; Sigma- Aldrich Chemical, St. Louis, MO) was dissolved at room temperature in sterile water and used within 1 h of preparation. Pregnant mice were injected IP with 600 mg/kg VPA on E8 at 10:00 hours (*n* = 22), mating date was defined as E-1. Control (Sham) animals were injected with the same volume of sterile water (*n* = 11)^12^.

### Tissue samples: brain, spinal cord

On embryonic day 17 fetuses were collected into three groups; exencephalic, internal control (nonexencephalic littermates from VPA-treated dams), and Sham (untreated dams). Whole brain and spinal cord samples dissected for RNA and protein expression analysis were dissected, snap frozen and stored at −80 °C. For histology and immunofluorescence, fetuses were fixed for 24 h in 4% paraformaldehyde, processed and embedded in paraffin. Samples for flow cytometry were dissected and processed following the standard protocol for the neural tissue dissociation kit (Miltenyi Biotec, Auburn, CA, USA); briefly, neural tissue was digested with papain at 37 °C with rotation using a gentle MACS Dissociator (Miltenyi Biotec, Auburn, CA, USA). Cell suspension was passed through 70 µm cell strainer (BD) and labeled for flow cytometry as described below.

### RNA extraction and RT-qPCR

Tissues were homogenized (IkaT10 basic Ultra-Turrax homogenizer, USA) in RLT buffer and RNA extracted using the RNeasy Plus Mini Kit (Qiagen Science, Hilden, Germany) following the manufacturer’s protocol. One microgram of RNA was reverse transcribed using RT2 First strand Kit (Qiagen Sciences, MD, USA) and then run on specific TaqManR Gene Expression Assays for: TPR53, CDKN2A, CDKN1A, RBL2, Caspase3 and Caspase9 (Applied Biosystems, Foster City, CA, USA) (Supplementary Table 1) and the 7500 Fast Real-Time PCR System. Samples were performed in triplicate. The relative expression fold change of mRNAs was calculated by the 2−ΔΔCt method on eight fetuses per group (*n* = 8).

### Protein extraction

Proteins were extracted from total fetal brain and spinal cord samples at E17 by sonication (Fisher Scientific, Pittsburgh, PA, USA) in N-PER buffer (Thermo Fisher Scientific, Rockford, IL, USA) + Proteinase K + PhosphoSTOP (Roche Diagnostics GmbH, Mannheim, Germany) and centrifuged at 10,000 rpm for 5 min. Protein concentration was assessed using the BCA-mini kit (Thermo Scientific, Rockford, IL, USA).

#### Western blot

Brain and spinal cord homogenates on six animals per group (*n* = 6) were loaded onto 4−12% acrylamide gels and transferred to PVDF membranes (Life Technologies, CA, USA). After blocking with BSA (5%) for 1 h, membranes were hybridized with primary antibody: anti-NeuN (1:1000, Abcam, Cambridge, MA, USA), anti-GFAP (1:1000, Dako, Carpinteria, CA, USA), anti-oligodendrocyte (1:250, Abcam, Cambridge, MA, USA), anti-Iba1 (1:1000, Wako, Richmond, VA, USA), anti-p21 (1:500, Abcam, Cambridge, MA, USA) and anti-ß-Actin (1:10,000, Abcam, Cambridge, MA, USA) overnight at 4 °C. Membranes were washed and hybridized with appropriate secondary antibody (1:10,000, Abcam, Cambridge, MA, USA) for 1 h at RT and visualized using SuperSignal WestPico Chemiluminescent substrate (Thermo Scientific, Rockford, USA). Protein expression was quantified by densitometry using ImageJ (National Institutes of Healt, Bethesda, MD) and normalized to ß-Actin.

### Histological analysis

Sagittal sections of the brain and spinal cord cross sections were fixed in formalin for 48 h and processed and embedded in paraffin blocks. Histology was performed on 5 μm sections from sagittal brain and spinal cord cross sections including the periventricular region, brain stem and cerebellum. Hematoxylin and eosin (H&E) staining was performed using a Varistain Gemini ES autostainer (Thermo Scientific). A 1:4 mixture of Mayer’s Hematoxylin (Lillie’s Modification) histological Staining Reagent (Dako Carpinteria, CA, USA) and Automation Hematoxylin Staining Reagent (Dako Carpinteria, CA, USA) was used with a 0.25% Eosin-Y (Richard Allen Scientific) for contrast.

### Immunofluorescence

Sections (5 µm) of fetal brain and spinal cord were dried, permeabilized with 0.1% Triton X-100 (Sigma Aldrich, St. Louis, MO, USA) in PBS and blocked for 1 h in 5% BSA at RT. Sections were incubated with primary antibody; anti-GFAP (1:1000, DAKO, Carpinteria, CA, USA), anti-Iba1 (1:1000, Wako, Richmond, VA, USA), anti-p21 (1:500, Abcam, Cambridge, MA, USA), anti-Cleaved Caspase 3 (1:500, Cell Signalling, Danvers, MA, USA), overnight at 4 °C in a humid chamber. Slides were washed and incubated for 1 h with Alexa Fluor 488 or 568 labeled secondary antibodies (1:1000, Life Technologies, Eugene, OR, USA) at RT in the dark. Slides were washed and mounted in mounting media with DAPI (SouthernBiotech, Birminghan, AL, USA).

### Neurotracer assay

Neurotracer assay (Life Technologies, Eugene, OR, USA) was performed on 5 µm sections of fetal brain and spinal cord following the manufacturers’ instructions to identify and visualize neurons.

### TUNEL assay

TUNEL assay (Roche, Diagnostics GmbH, Mannheim, Germany) was performed following the manufacturers’ instructions on brain and spinal cord at E17 to analyze apoptosis/cell death^13^.

### Flow cytometry with Annexin V (AV) and Propidium Iodide (PI)

Phosphatidyl-serine is located on the inner part of the plasma membrane facing the cytosol in normal, live cells, but during apoptosis it is translocated to the cell surface and can be labeled with Annexin V (AV). The vital dye propidium iodide (PI) was used to determine cell membrane injury in the single-cell suspension from neural tissue. The percentage of cells labeled as PI + AV+ (late apoptosis), PI + AV− (necrosis), PI − AV+ (early apoptosis) and PI − AV− (live cells) were quantified following flow on an LSR III (BD Biosciences, San Jose, CA, USA) and FlowJo (FlowJo LLC, Ashland OR, USA) analysis software.

### Statistical analysis

Results are expressed as means ± standard error (SE) for relative gene expression (2-ΔΔCt) and means ± standard deviation (SD) for protein expression. Inter-group comparisons were performed with the unpaired Student’s *t* test considering a *P* value of <0.05 significant. GraphPad Prism 7 (GraphPad Software Inc., La Jolla, CA, USA) package was used for statistical calculations.

## Results

### Loss of layer organization in VPA-induced exencephalic fetuses

**Fig. 1:**
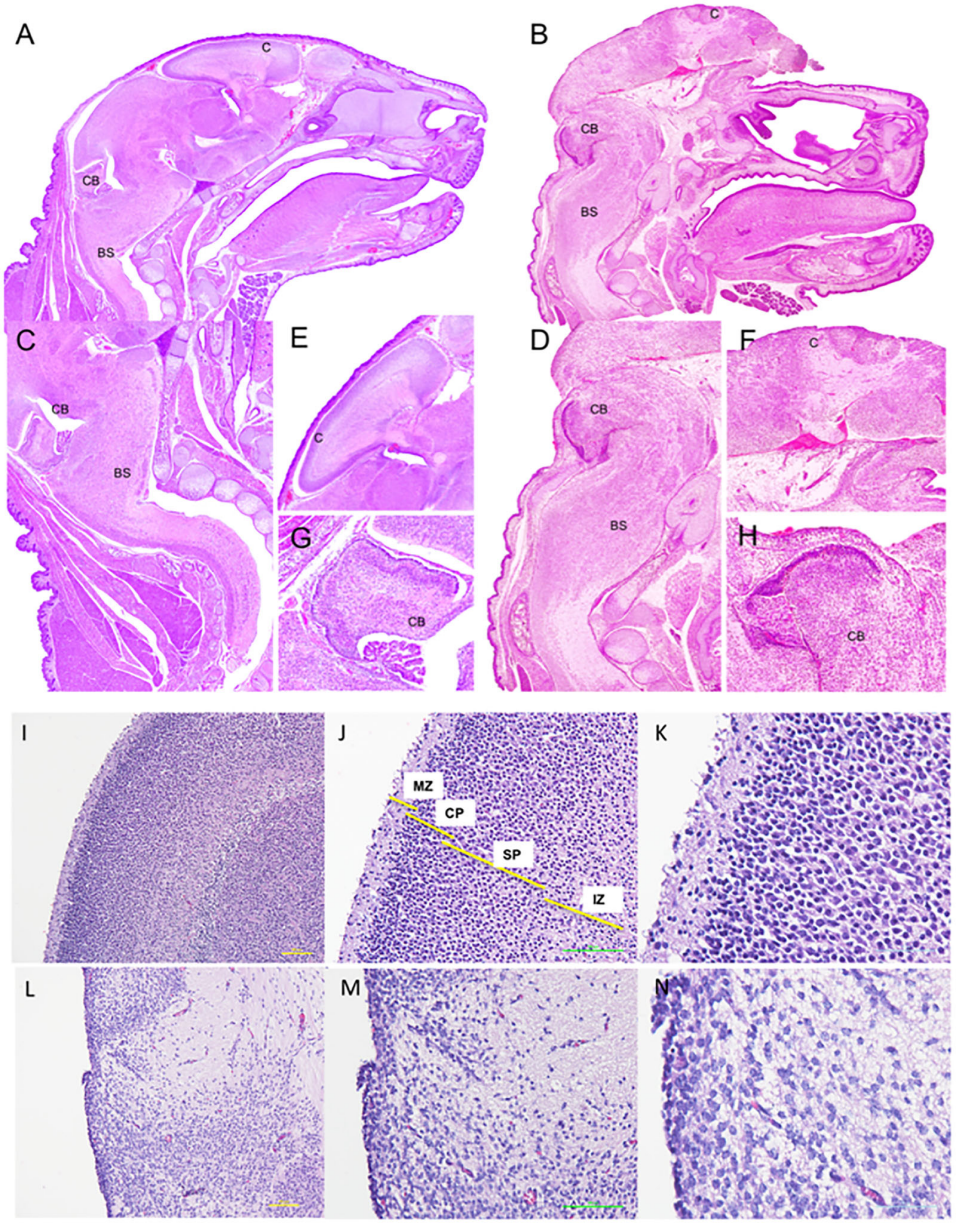
Brain maldevelopment in VPA-induced exencephaly. Sagittal section of normal (**a**) and exencephalic (**b**) fetal head at E17 (4× H&E); Cerebellum and brain stem in normal fetuses (**c**) and hypertrophic brain stem in exencephaly fetuses (**d**) (10×, H&E); normal structured cortex (**e**) and brain cortex with no structures in exencephaly (**f**) (20×, HE) and cerebellum with cortical layers and circumbulutions in normal fetus (**g**) and nonstructured mass in exencephalic fetuses at E17 (**h**) (20×, HE). C cortex, CB cerebellum, BS brain stem. Normal cortical layer organization in Sham (**i**–**k**) and loss of any cellular layer organization in exencephaly (**l**–**n**) (10×, 20× and 40×, HE). MZ marginal zone, CP cortical plate, SP sub plate, IZ intermediate zone

Twenty-two percent of the fetuses developed exencephaly following maternal administration of VPA. We observed typical alterations seen in exencephalic fetuses with absent frontal, parietal and occipital bones (Fig. 1b).

At E17 exencephalic fetuses demonstrated a lack of structural organization in areas such as the olfactory bulb, hippocampus, dental gyrus, and septal complex, with evidence of collapsed ventricles (Fig. 1b, f). The brain stem was hypertrophic (Fig. 1b, f) and the cerebellum atrophied with no circumvolutions nor cortical layer organization (Fig. 1d, h). Control fetuses from nonaffected littermates and those from sham mothers demonstrated normal brain development and structural organization (Fig. 1a–g).

**Fig. 2:**
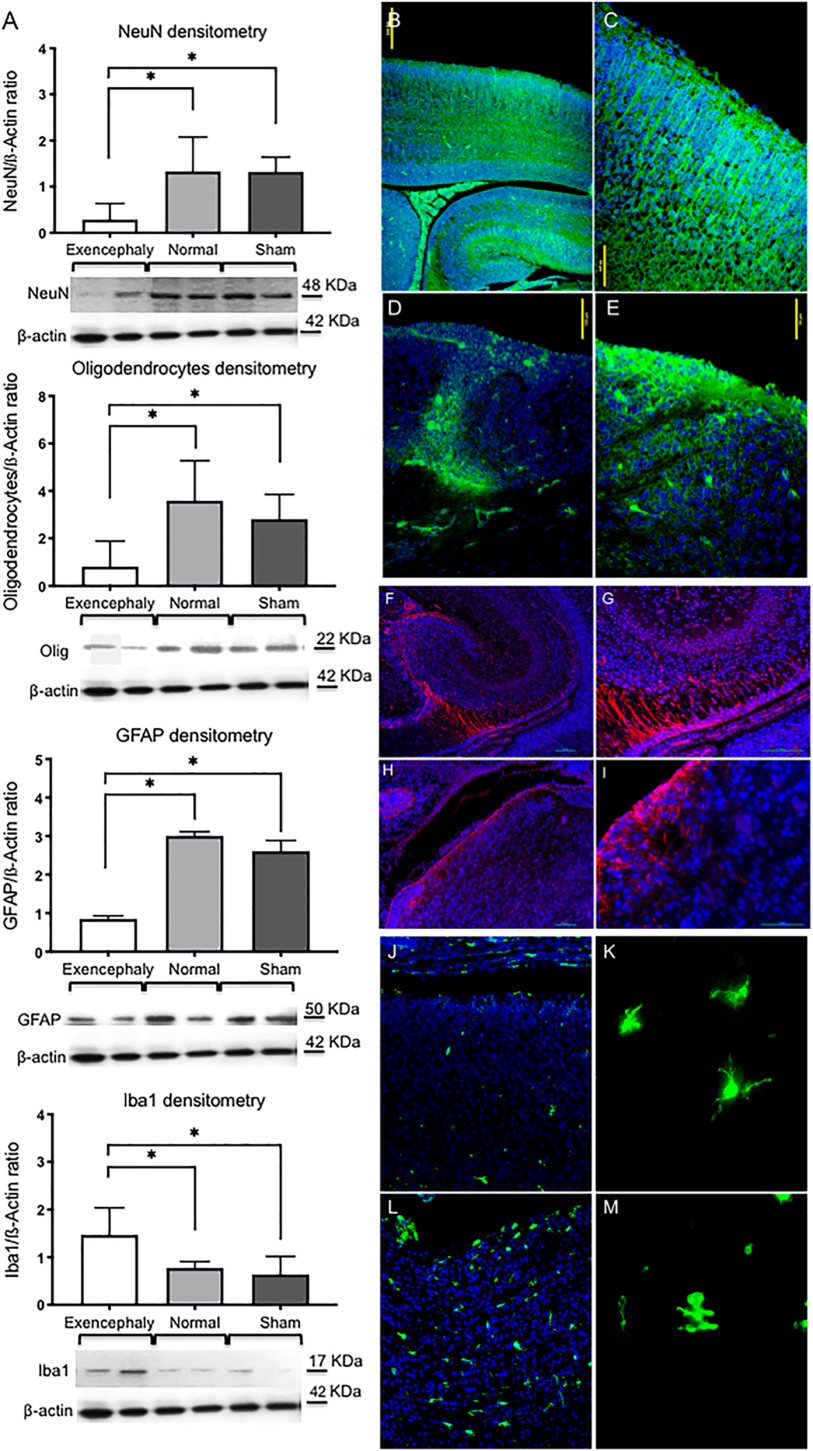
Downregulation of neurons, astrocytes, oligodendrocytes and upregulation of microglia in the exencephalic brains. Western blot analysis showed a clear reduction in protein expression levels in the brain of **a** neurons (NeuN), astrocytes (GFAP) and oligodendrocytes. Also, an increase of the protein expression of microglia (Iba1) in brain lysates from exencephaly vs. normal and vs. Sham group (*p < 0.05) (*n* = 6−8). Neuronal marker staining (Neurotracer) showed normal structured brain (**b**) with normal layer organization in the cortex (**c**) at E17 in normal fetuses at E17 compared with no neuronal organization in exencephalic fetuses (**d**, **e**). GFAP+ cells expressed in the ventricular zone, dentate gyrus and fimbria in normal brain at E17 (**f**, **g**). GFAP+ cells are restricted around the ventricle in exencephalic mice (**h**, **i**). Neurotracer (green), GFAP (red) and DAPI (blue) (20× and 40×). Microgliosis at the external layers of the brain exposed to the amniotic fluid with activated morphology in exencephalic brain with activated morphology (**l**, **m**) compared with less expression in normal fetuses with resting phenotype (**j**, **k**). Iba1 staining (green) (20× and 100×) (*n* = 6–8)

The exencephalic brain cortex showed no layered cell organization (marginal zone, cortical plate, sub plate and intermediate zone) (Fig. 1l–n) compared to littermate controls and sham (Fig. 2a–c)^14^. Derangement of neurons was observed in the gray matter with neuronal cells randomly arranged from the superficial molecular layer to the deeper cortical layer, without laminar organization (Fig. 1i, k).

### Reduction of neuronal and glial protein expression in exencephalic fetuses

The brains of the exencephalic fetuses showed lower protein expression of NeuN, GFAP, Oligodendrocyte compared to control and sham group (*p* < 0.005); in contrast, exencephalic fetuses showed increased Iba1 protein expression in the brain compared to sham and littermate controls (Fig. 2a). Immunofluorescence for Neurotracer in the brain corroborates these results (Fig. 2b–e). The exencephalic fetuses showed dispersed neuronal distribution with no structural organization or visible cortical distribution (Fig. 2d, e) compared with control and sham fetuses at E17 in which the outline of the dentate gyrus, including the hippocampal fissure, was clearly identifiable (Fig. 2b) along with the cortical projections (Fig. 2c).

At embryonic day 17 in normal brain development, GFAP expression is limited to cells in the ventricular zone (VZ), suprafimbrial region, subpial region between the fimbria and the dentate gyrus (Fig. 2f, g)^15^. These astrocytes (GFAP positive cells) are the migrating cell population that contribute to the formation of the dentate granule cell layer, but not the pyramidal cell layer as Seki et al. previously described^16^. In exencephalic fetuses at E17 the astrocytes (GPAP positive cells) are expressed in the layers in contact with the ventricles as a potential VZ but lack a well-defined pattern (Fig. 2h, i).

In control littermates and sham fetuses, microglial expression of Iba1 is limited to the brain cortex at E17 (Fig. 2j) and Iba1-positive cells showed a resting phenotype, with extended projections (Fig. 2k). In contrast, in the exencephalic fetuses there was an increase in numbers of microglia demonstrating activated morphology (Fig. 2l, m) and these were mainly expressed in the layers exposed to amniotic fluid.

### Detection of necrosis, apoptosis and senescence in VPA-induced exencephalic fetuses

**Fig. 3:**
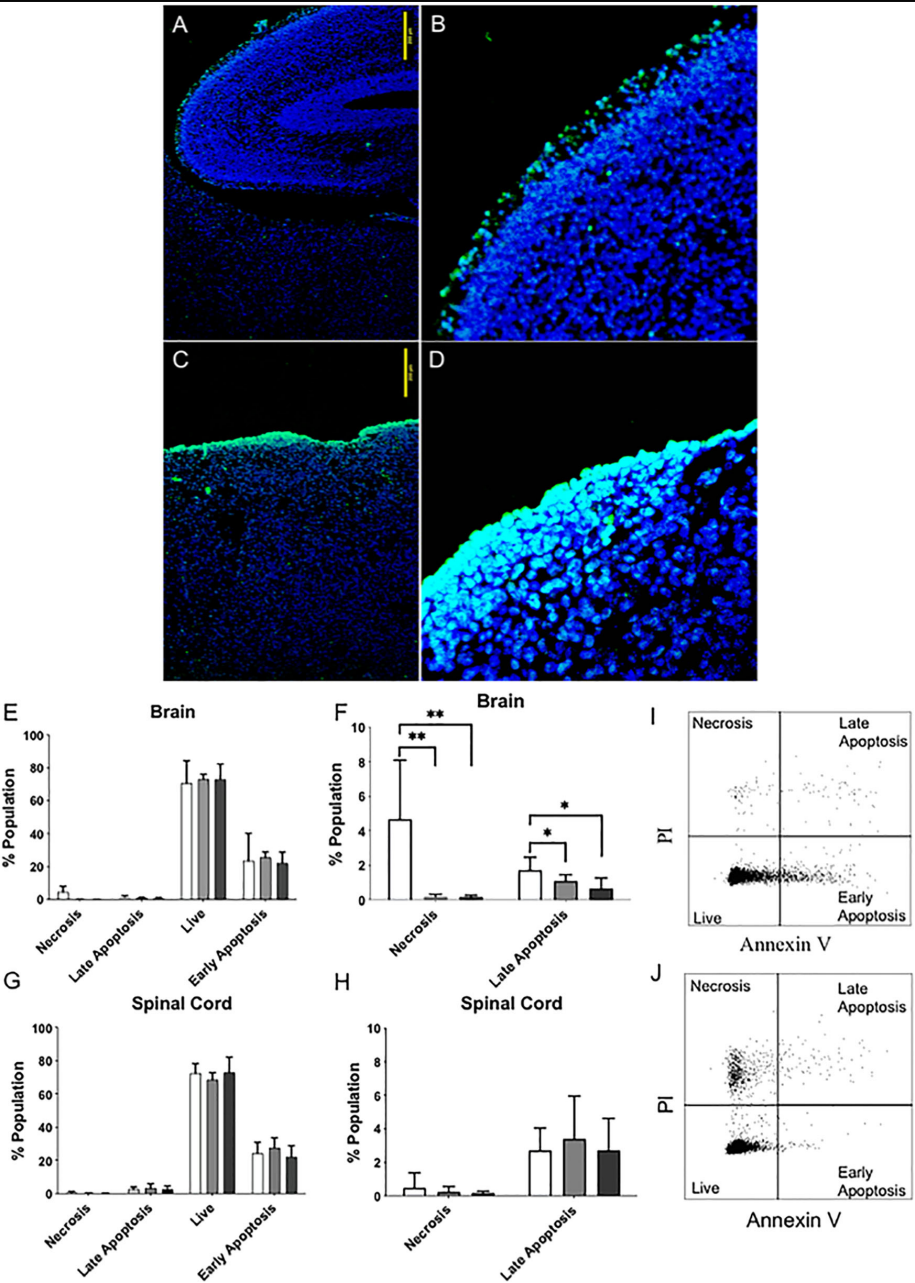
Increased necrosis/apoptosis in exencephaly brains VPA-induced mice. Brain sections of control fetuses at E17 (**a**, **b**) with minimal TUNEL-positive cells (green) and exencephalic brains (**c**, **d**) with TUNEL-positive cells in exposed brain to the amniotic fluid (**a** and **c** 40×) (**b** and **d** 40×). The apoptosis/necrosis analysis by flow cytometry with PI and Annexin V in the brain (**e**, **f**) and spinal spinal cord (**g**, **h**), % population mean (±SD) (*n* = 6). Exencephalic brains show higher % of cells PI + AV+ (necrosis) and PI − AV+ (late apoptosis) (**f**, **j**) (*p < 0.05, **p < 0.001) than control and sham fetuses (**f**, **i**). No differences seen in the spinal cord (**g**, **h**) (*n* = 6–8)

Following VPA exposure and nonclosure of cranial bones, neural tissue remains exposed to the amniotic fluid. Exencephalic fetuses at E17 exhibited necrosis/cell death in the cell layer exposed to amniotic fluid (Fig. 3c, d). In controls, no cells in the inner brain and minimal cells in the outer layers stained positive with TUNEL (Fig. 3a, b)

The percentage of necrotic and late apoptotic cells, (AV−/PI+ and AV+/PI+) were significantly increased in exencephalic brains (Fig. 3f, j) compared to control and sham fetuses (Fig. 3f, i) (*p < 0.05). No changes were seen in the number of live, AV−PI−, and early apoptosis, AV+/PI events (Fig. 3e). Furthermore, no differences were seen in the cell populations of the spinal cord (Fig. 3g, h).

### Overexpression of intrinsic apoptosis pathway in the brain and spinal cord of VPA-induced exencephalic fetuses

**Fig. 4:**
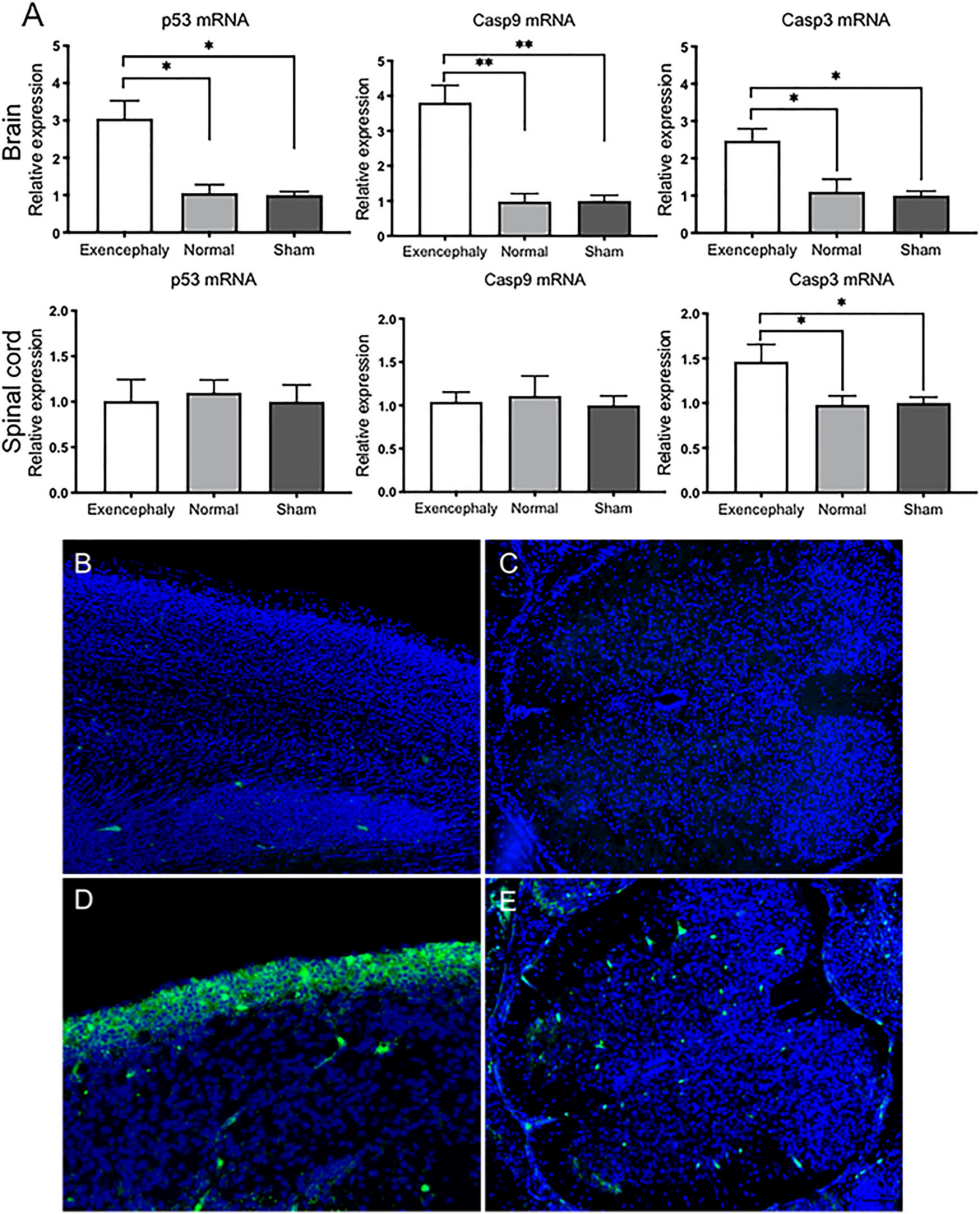
Exencephaly activates apoptotic genes in the brain and spinal cord. Relative gene expression for intrinsic apoptotic genes (p53, Caspase3 and Caspase9) in brain and spinal cord (**a**). Exencephalic fetuses at E17 showed upregulation of p53, Caspase3 and Caspase9 in the brain for and Caspase3 in the spinal cord vs. Sham and Control group (*p < 0.05, **p < 0.001). Caspase 3-positive cells (green) in the region exposed to the amniotic fluid in exencephalic brains (**d**) and scattered staining of active Caspase 3 in the spinal cord (**e**). No Caspase3 is seen in the brains nor spinal cords from normal fetuses at E17 (**b**, **c**) (*n* = 6–8)

Exencephalic fetuses had increased brain expression of the intrinsic apoptosis genes p53, Caspase 9 and 3 but only showed increased Caspase 3 expression in the spinal cord (*p < 0.05, Fig. 4a). Activated Caspase 3 was expressed in the layers of the neural tissue exposed to amniotic fluid in exencephalic fetuses (Fig. 4d) and in the nonexposed spinal cord (Fig. 4e) when compared with normal and sham neural tissue (Fig. 4b, c).

### Local and diffuse overexpression of cdkn1a (p21)-Retinoblastoma (Rbl2) in the brain and spinal cord of VPA-induced exencephalic fetuses

**Fig. 5:**
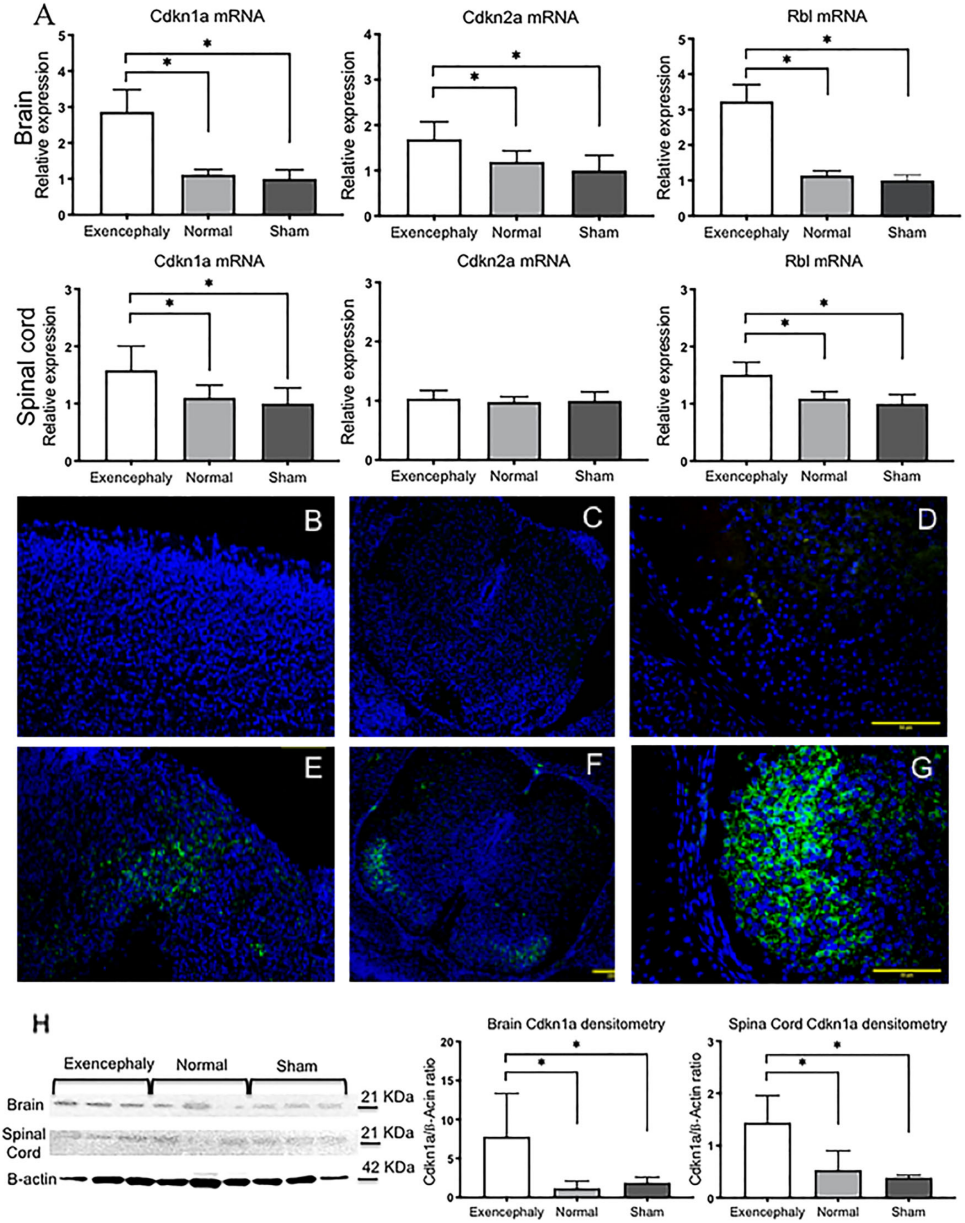
Senescence via p21 in VPA-induced exencephalic fetuses at E17. The relative expression of senescence genes (p21, p16 and Rbl2) in brain and spinal cord (**a**) of exencephalic fetuses at E17 showed upregulation of p21-p16-Rbl2 senescence pathway in the brain and p21-Rbl2 in the spinal cord. Exencephaly vs. Sham and Control groups (*p < 0.05). p21-positive cells (green) in the brain are scattered in clustered cell groups in exencephalic brains (**e**) and expressed in the lamina I and II of the spinal cord (**f**, **g**). No expression of p21 was seen in normal brains nor spinal cords at E17 (**b**–**d**). Relative protein expression of p21 by western blot in brain and spinal cord tissue from normal and exencephalic fetuses at E17 of gestation (**h**) (*p < 0.05) (*n* = 6–8)

Exencephalic fetuses also exhibited increased gene expression of senescence-related genes p21, p16 and Rbl2 in the brain and p21 and Rbl2 in the spinal cord compared to the control and sham group (*p < 0.05, *n* = 6, Fig. 5a). This increase was reflected at the protein level (Fig. 5h) and localized by immunofluorescence to scattered neurons in the brain (Fig. 5e). P21-positive neurons were also identified in the lamina I and II in the posterior horn of the spinal cord (Fig. 5f, g). No p21 expression was found in the brain (Fig. 5b) nor the spinal cord of control and sham fetuses (Fig. 5c, d).

## Discussion

In this study, we described the activated mechanisms of the central nervous system in the valproic acid-induced murine model of exencephaly. We analyzed changes on day 17 of gestation in the brain and the spinal cord to characterize the effect of direct exposure and indirect exposure to amniotic fluid. Focusing on day 17 we characterized the alterations in the CNS prior to total destruction of the brain tissue (anencephaly) to understand the mechanisms involved in the pathophysiology of this model^9^. This point in gestation is crucial to understand the neurodegenerative and maldevelopment mechanisms of the brain exposed to amniotic fluid as well as the indirect effect on neurodegeneration in other further areas of the CNS such as the spinal cord, which is not exposed to the chemical and mechanical actions of the amniotic fluid. Similar effects occur in other open neural tube defects like open spina bifida in which the spinal cord exposure to amniotic fluid induced progressive neurodegeneration with neuroinflammatory response^3^.

VPA causes lack of skull protection to the developing brain, which generates severe disruption to the laminar architecture of the cerebral cortex^17^. We found no structural tissue organization in exencephalic fetuses, and the brain protrudes like a mass of tissue when exposed to the aggressive AF environment. Neurons are not organized in the expected layers and axonal projections are not identifiable. There is variability among fetuses in the shape of the exposed brain and the degree of neural tissue loss. Furthermore, architecture also is disrupted in regions with reduced cellular density.

We found a reduction in the total protein expression of neurons, oligodendrocytes and astrocytes that corroborate with previous studies using the same teratogen^18^. In our model, GFAP expression in migrating astrocytes is restricted to the area around the ventricles in the exencephalic fetuses. GFAP expression in the developing mouse brain follows the basic progression of developing radial glia and astrocytes. GFAP expression can be detected earlier in normal mice with the radial glia genesis around the telencephalic VZ^19^ and then in the fimbria^20,21^. At E17 in normal mice we observed astrogenesis which was not seen in the exencephalic fetuses. Exencephalic mice at E17 resembled normal mice at E9.5−E11 with expression of only the first radial glia^22^.

Using flow cytometry, we evaluated necrosis and apoptosis in the neural tissue of the brain directly exposed to the amniotic fluid and indirectly in the spinal cord. Analyzing the early, late apoptotic and necrotic cells from the CNS tissue by flow cytometry and the histological images demonstrate the effect of the in utero environment to the exposed cell layers^2,23^. This exponential chemical action due to enzymes present in the amniotic fluid increases during late gestation^24^ and physical action by friction with the amniotic membranes^2^ could be the mechanism inducing apoptosis in the external layers of the brain but not in the protected areas, such as the spinal cord. In those areas directly exposed to the amniotic fluid, we observed a neuroinflammatory response involving microglia activation. While microglia are the resident macrophage population of the CNS, their function is more than being the first immune sentinel cell, they also contribute to CNS homeostasis and cell proliferation. However, over reactive microglia leads to neural degeneration in neural tube defect injured tissues^3^.

The continued cell death in the brain layers exposed to amniotic fluid lead to an indirect disruption to the spinal cord. These alterations were detected by upregulation of the intrinsic apoptotic genes p53 and Caspase 9 and 3 genes in the exposed brain tissue but also in the spinal cord of the fetuses with exencephaly. Apoptotic cells were detected in the external layers of the brain exposed to the amniotic fluid. This apoptosis could underlie the functional locomotor alterations with abnormal motor behavior that anencephalic fetuses show after birth^25,26^. Also, the activation of these apoptotic markers in the spinal cord could determine diffuse progression of the corticomotor deficits and the loss of reflex^5^.

Both growth arrest and apoptosis are associated with the induction of p53, particularly in response to DNA damage or other modes of cellular stress^27^. We observed upregulation of CDKN1A (p21) and retinoblastomas protein (Rbl2) in the exencephalic mice in the brain; however, there was no specific pattern of cells expressing p21 in the brain which may be due to the lack of cytoarchitecture and structural organization. It is possible these p21-positive neurons are from the hippocampus as has previously been described^28^.

Interestingly, p21-positive cells were found in the lamina I and II at the Subtantia Gelatinosa of the dorsal horn in the absence of activation of apoptotic genes (p53, Caspase 3 or Caspase 9) in that region. These results suggest that the somatotopically areas are in a senescent state probably due to the loss of the somatosensory circuits in the brain. The loss of the neurons in the brain, including the somatosensory brain areas, and the alteration of motor responses by the progressive and continued abrasion of the brain layers by the amniotic fluid plus the internal disruption affect the sensory neurons in the dorsal horn^29^. Further experiments with neuronal tracers are needed to reveal if the p21-positive cells in the brain and the spinal cord are part of the same neuronal circuits.

## Conclusion

Exencephaly is one of the leading causes of fetal and neonatal mortality as the most extreme open neural tube defect. There are currently no treatments, and the etiology and progression of this lethal malformation are poorly investigated. The current study analyzing CNS disruptions in exencephalic fetal mice demonstrates neurodegeneration due to extrinsic local cell death, but also by intrinsic apoptosis and senescence in both the brain and spinal cord. This local and diffuse cell degeneration may contribute to the devastating neurological alterations seen at birth.

## Supplementary information

TaqMan probes for gene expression assay

## Data Availability

The authors declare that the data supporting the findings of this study are available within the article and its supplementary information.
