# Supplementary material for: Cell necrosis, intrinsic apoptosis and senescence contribute to the progression of exencephaly to anencephaly in a mice model of congenital chranioschisis
Source: Cell Death Dis. 2019 Sep 26;10(10):721. doi: 10.1038/s41419-019-1913-6 (PMC6763477; doi:10.1038/s41419-019-1913-6)
Supplement: Supplementary file 1 — TaqMan probes for gene expression assay [file 41419_2019_1913_MOESM1_ESM.docx]

| **mRNA** | **Name** | ***Assay code number** |
| --- | --- | --- |
| TPR53 | Transformation protein related 53 | Mm01198158 |
| CDKN2A | Cyclin-dependent kinase inhibitor 2A | Mm0049449_m1 |
| CDKN1A | Cyclin-dependent kinase inhibitor 1A | Mm04205640_g1 |
| RBL2 | Retinoblastoma-Like 2 | Mm01242468 |
| Casp3 | Caspase-3 | Mm01195085 |
| Casp9 | Caspase-9 | Mm00516563_m1 |

**Table1**. TaqMan probes for gene expression assay

*Probes code assay from Applied Biosystems, Foster City, CA, USA
